# Dental Educators' Stress and Wellbeing in the Workplace—An International Perspective

**DOI:** 10.1111/eje.70049

**Published:** 2025-09-10

**Authors:** Shannu Kohli Bhatia, Ruby Long, Sviatlana Anishchuk, Damian J. J. Farnell, Morag Powell, Michael G. Botelho

**Affiliations:** ^1^ School of Dentistry Cardiff University Cardiff UK; ^2^ Trinity College Dublin The University of Dublin Dublin Ireland; ^3^ Peninsula Dental School University of Plymouth Plymouth UK; ^4^ University of Hong Kong Hong Kong China

**Keywords:** dental educators, dental staff, educator barriers, stress, wellbeing

## Abstract

**Introduction:**

Supporting wellbeing of staff involved in dental education is vital to ensure the safe effective delivery of the curriculum and training of the dental workforce. There are only a limited number of studies on the stress and wellbeing of staff involved in dental education and the barriers they face in engaging with any wellbeing services provided. To plan strategies for the promotion of staff wellbeing, it is important to identify these and the barriers faced by staff. The aim of this study is to determine the stress and wellbeing of the staff involved in dental education and identify any barriers they face in accessing wellbeing services.

**Methods:**

An online cross‐sectional survey was conducted to investigate the wellbeing and stress of staff involved in dental education in institutions associated with the Association of Dental Education in Europe, using two validated survey instruments: the Warwick‐Edinburgh Mental Wellbeing Scale (WEMWDS) and the Perceived Stress Scale (PSS). In addition, staff demographics and barriers to accessing any wellbeing services were identified.

**Results:**

A total of 247 participants responded. The mean WEMWDS score was 49.0 (95% CI = 47.9–50.1; SD = 8.7) and the mean PSS score was 18.1 (95% CI = 17.3–19.0; SD = 6.7), with 68.0% reporting moderate and 8.5% high levels of perceived stress. Year of birth and work role were statistically significant for the primary outcome. Over 50% of respondents who needed support did not access the available services, citing several barriers, including lack of awareness (15%), uncertainty about the effectiveness of services (20.6%) and time constraints (22.3%).

**Conclusion:**

Staff involved in dental education report higher stress and lower wellbeing than the general population. Those in the younger age group or involved in job roles such as research or clinical teaching are more affected. Staff face multiple barriers to accessing wellbeing services and are more likely to seek help from senior colleagues. It is vital that educational institutions establish strategies to promote the wellbeing of their staff members and improve access to services.

## 1 Introduction

Wellbeing is a positive state experienced by individuals and societies that encompasses quality of life and the ability of people and societies to contribute to the world with a sense of meaning and purpose [1]. It has also been defined as a state of complete physical and mental health that is characterised by high‐quality social relationships. Everyone working in education should have the opportunity to enjoy the highest possible standard of wellbeing and mental health [2]. Stress can adversely affect one's wellbeing. Stress is described as the state that occurs when a person encounters events perceived as endangering or threatening to their ability to cope and deal with the situation [3, 4]. Living in our current society, stress is arguably a part of our daily lives. However, when it exceeds an individual's ability to cope, it can have a detrimental impact on one's mental health and wellbeing and, if remains unchecked, may progress to burnout. Burnout may manifest as psychological exhaustion, loss of feeling and concern, depersonalisation and reduced productivity and capability [5]. The aim of this study is to determine the stress and wellbeing of the staff involved in dental education and identify any barriers they face in accessing wellbeing services.

Dentistry is considered to be a stressful occupation with high levels of stress, burnout and psychological distress [6, 7]. The stressors that dental health care professionals are under include working under time and academic pressures, coping with difficult patients, medical emergencies and dissatisfied patients, management/staff issues, clinical and nonclinical paperwork and fear of complaints/litigation [8, 9]. Stress and burnout not only impact the mental health and wellbeing of the individuals but can also have a detrimental impact on the profession in terms of absenteeism and compromised patient care and diminished professional standards [10]. Studies have shown that stress and anxiety can significantly impact individuals' clinical performance. A strong link between stress and impaired surgical competence and communication has been reported [11]. Studies have found a significant association between burnout and poor patient safety outcomes, compromised work performance and absenteeism [12, 13, 14, 15]. In addition, stress increases the risk of developing mental health conditions, such as depression [8]. This remains especially important as dentists, dental therapists and hygienists have reported that they experience low levels of mental wellbeing compared to the general population, and the stress they experience is reported to be predominantly workplace centred [8].

Mental health and wellness of the dental team is critical in maintaining and retaining a healthy workforce. This holds true for dental health professionals involved in dental education, although there is limited literature on their wellbeing and stress. A post‐pandemic study [16] revealed that females and younger dental faculty members reported statistically significantly lower levels of wellness and mental wellbeing, fulfilment, higher levels of burnout and a significantly lower work‐life balance. Dental educators will not be able to deliver their role and to support their students effectively if their own wellbeing is compromised. This is likely to affect the delivery of the curriculum and training of the future dental workforce.

The role of improved wellbeing in enhancing the working environment is well‐documented. According to the General Dental Council's rapid evidence assessment on mental health and wellbeing in dentistry, the dental sector needs to prevent and address professionals' mental health issues at every stage in their career journey—from education through into the workplace and through continuing professional development [7]. Educational establishments have a moral and ethical responsibility for supporting their staff effectively and for promoting their wellbeing and resilience. Identification and tackling the causes of poor wellbeing and resilience and offering appropriate, timely support to staff will enable them to improve their working environment, maximise their potential to deliver better quality patient care, and improve their quality of life. Investing in mental health and wellbeing—including anxiety and stress management—can help to avoid burnout in members of the workforce and maintain good outcomes for patients. Dental schools have a duty of care to their staff. To provide a safe and effective environment, organisations must protect their staff against burnout and emotional exhaustion through targeted intervention and prevention strategies [13]. The good mental health and wellbeing of dental educators is vital to ensure safe effective delivery of the curriculum and training of the dental workforce. Anecdotally, wellbeing services to support mental health and wellbeing are often provided by educational establishments. However, the uptake among staff remains poor. There are limited studies on the stress and wellbeing of dental staff and the barriers they face in engaging with the wellbeing services that are available to them. To allow planning of strategies for promotion of staff wellbeing, it is important to identify these.

This is the first of a series of papers that respectively report: (1) Stress and Wellbeing levels of the staff involved in dental education and barriers faced in accessing any wellbeing services. (2) Qualitative exploration of dental educators' perceptions and suggestions on improving their workplace wellbeing. (3) Qualitatively explore factors affecting the wellbeing of dental educators. The aim of the study presented in this paper is to determine the stress and wellbeing of the staff involved in dental education and identify any barriers they face in accessing wellbeing services.

## 2 Methods

### 2.1 Study Design

This cross‐sectional survey was conducted to investigate the wellbeing and stress levels of staff involved in dental education in dental schools/institutions associated with the Association of Dental Education in Europe (ADEE) during 2022/23. Ethical approval was obtained from the Cardiff University Ethics committee (reference number: 2315).

### 2.2 Data Collection

A convenience sample of all ADEE member dental school staff were invited to participate voluntarily. The survey was distributed electronically via the ADEE online newsletter. The survey link remained active for 3 months, accompanied by a participant information sheet and consent form. The data collection was carried out via Microsoft forms and was anonymous.

### 2.3 Survey Instrument

A survey was developed, pilot tested and included previously two validated instruments. It consisted of three parts:

1. Demographic Information: Participants provided details about their age range, gender, years of experience, job title, employment type and educational background.
2. Wellbeing Assessment: The Warwick‐Edinburgh Mental Wellbeing Scale (WEMWBS) a short and psychometrically robust scale that was used to measure overall mental wellbeing. The WEMWBS is a validated 14‐item scale that has been shown to be responsive to change and is suitable for various populations and settings [17] (Appendix A).
3. Stress Assessment: The Perceived Stress Scale (PSS‐10) was used to assess perceived stress levels. The PSS‐10 is a validated 10‐item scale that measures the degree to which situations are perceived as stressful, uncontrollable and overwhelming [18] (Appendix B).

In addition to the wellbeing and stress assessment tools mentioned above, the survey also included three closed‐ended questions identifying the availability of support services within the workplace and barriers faced in accessing them, and an open‐ended question asking participants views on how their dental school/University can make changes to help improve staff wellbeing. These questions explored the existence of wellbeing policies, the availability of support services and participants' experiences with seeking and using these services. The responses were analysed by calculating the scores and statistical analysis was carried out.

### 2.4 Statistical Analysis

Descriptive statistics, graphical methods and frequencies were used to explore the data initially. An exploration of the relationship between the primary outcomes (WEMWBS and PSS scores) was carried out via a simple scatter plot, a quadratic line fit and via Spearman's correlation coefficient. Normality for the primary outcomes was assessed via histograms and normal plots (WEMWBS and PSS scores); both variables were found to be normally distributed. Variances with each group for each factor were equivalent for both WEMWBS and PSS scores via tests of homogeneity of variances (*p* > 0.05). Univariate statistical tests (i.e., one‐way ANOVA) were employed initially, where Tukey's post hoc test was used to identify significant differences between groups. Interactions between all factors were then explored via two‐way ANOVA and via graphical methods, although these were found not to be either strong or significant (*p* > 0.05). A mixed‐model analysis with main effects only was carried out as a final exploration and confirmation of results. Fixed effects were assumed on all factors, except geographic region, which was set to be random. All statistical analyses were carried out using SPSS V29.

## 3 Results

A total of 247 participants responded to the survey *n =* 247. Subject characteristics are shown in Table 1. The most common years of birth were between 1981 and 1996 (*n =* 118; 47.8%), followed by 1965 and 1980 (*n =* 98; 39.7%). Respondents were predominantly female (*n =* 167 subjects out of 247; 67.6%) and from the UK (*n =* 171; 69.1%). Most subjects were married (*n =* 171; 61.1%) or single (*n =* 74; 30.0%) and in full‐time employment (*n =* 146; 59.1%). The most common main working role was clinical teaching (*n =* 109; 44.1%), which was followed by mainly clinical (*n =* 55; 22.3%).

**TABLE 1:** Subject characteristics.

**Year of birth**	1946–1964	1965–1980	1981–1996	1997–2012		
*n*	27	98	118	4		
Percentage	10.9	39.7	47.8	1.6		
**Gender**	Female	Male	Other	Prefer not to say		
*n*	167	78	1	1		
Percentage	67.6	31.6	0.4	0.4		
**Geographic region**	UK	Other	Europe^a^	Ireland	Australia	
*n*	171	14	15	35	12	
Percentage	69.2	5.7	6.1	14.2	4.9%	
**Marital status**	Civil partnership	Divorced	Married	Single		
*n*	11	11	151	74		
Percentage	4.5	4.5	61.1	30.0		
**Employment**	Full time	Part time 2 days a week or more	Part time < 2 days a week			
*n*	146	64	37			
Percentage	59.1	25.9	15.0			
**Role**	Administration	Clinical	Teaching (Clinical)	Other	Research	Teaching (non‐clinical)
*n*	32	55	109	21	15	15
Percentage	13.0	22.3	44.1	8.5	6.1	6.1

^a^ Europe: excluding the UK and Ireland.

Evaluated over all subjects, the mean WEMWDS score was 49.0 (95% CI = 47.9–50.1; SD = 8.7) and the mean PSS score was 18.1 (95% CI = 17.3–19.0; SD = 6.7). 58 out of 247 subjects (23.5%) had low levels of perceived stress, 168 (68.0%) had moderate levels of perceived stress and 21 (8.5%) had high levels of perceived stress. The numbers and percentages (with respect to the overall sample size of 247) responding to each item in the WEMWBS questionnaire are shown in Table 2.

**TABLE 2:** Numbers and percentages (with respect to the overall sample size of 247) responding to each item in the WEMWBS questionnaire.

		Optimistic about the future	Feeling useful	Feeling relaxed	Interested in other people	Had energy to spare	Dealing with problems well	Thinking clearly	Feeling good about myself	Feeling close to other people	Feeling confident	Able to make up my own mind about things	Feeling loved	Interested in new things	Feeling cheerful
None of the time	*n*	6	2	14	4	31	5	2	6	3	6	2	11	6	3
	%	2.4	0.8	5.7	1.6	12.6	2.0	0.8	2.4	1.2	2.4	0.8	4.5	2.4	1.2
Rarely	*n*	21	15	65	17	68	11	17	23	37	27	16	38	22	18
	%	8.5	6.1	26.3	6.9	27.5	4.5	6.9	9.3	15.0	10.9	6.5	15.4	8.9	7.3
Some of the time	*n*	83	56	92	56	90	83	68	89	98	79	59	75	65	82
	%	33.6	22.7	37.2	22.7	36.4	33.6	27.5	36.0	39.7	32.0	23.9	30.4	26.3	33.2
Often	*n*	109	127	65	126	46	127	137	107	86	104	123	79	96	114
	%	44.1	51.4	26.3	51.0	18.6	51.4	55.5	43.3	34.8	42.1	49.8	32.0	38.9	46.2
All of the time	*n*	28	47	11	44	12	21	23	22	23	31	47	44	58	30
	%	11.3	19.0	4.5	17.8	4.9	8.5	9.3	8.9	9.3	12.6	19.0	17.8	23.5	12.1

The percentage of subjects that responded ‘Rarely’ or ‘None of the time’ for the PSS‐10 questionnaire was generally < 10% for most items. However, the percentages of subjects responded ‘Rarely’ or ‘None of the time’ to the items relating to feeling relaxed, having energy to spare, feeling close to other people and feeling loved were much higher, namely, 32.0%, 40.1%, 16.2% and 19.2%. The numbers and percentages responding to each item in the PSS questionnaire is shown in Table 3. Elevated levels of perceived stress are in evidence from this table for some subjects. For example, this shown by the percentages of subjects who responded with ‘Never’ or ‘Almost Never’ for the positive scales or ‘Fairly Often’ or ‘Always’ for the negative scales for the items: upset because of something that happened unexpectedly (*n* = 66, 26.7%, negative scale), unable to control the important things in your life (*n* = 71, 28.7%, negative scale), unable to cope with all the things that you had to do (*n* = 62, 25.1%, negative scale), on top of things (*n* = 63, 25.5%, positive scale), angered because of things that were outside of your control (*n* = 70, 28.3%, negative scale), and difficulties were piling up so high that you could not overcome them (*n* = 61, 24.7%, negative scale).

**TABLE 3:** Numbers and percentages (with respect to the overall sample size of 247) responding to each item in the PSS questionnaire.

		Been upset because of something that happened unexpectedly?	Felt that you were unable to control the important things in your life?	Felt nervous and ‘stressed’?	Felt confident about your ability to handle your personal problems?	Felt that things were going your way?	Found that you could not cope with all the things that you had to do?	Been able to control irritations in your life?	Felt that you were on top of things?	Been angered because of things that were outside of your control?	Felt difficulties were piling up so high that you could not overcome them?
Scale		Negative	Negative	Negative	Positive	Positive	Negative	Positive	Positive	Negative	Negative
Never	*n*	20	27	11	4	3	18	1	7	18	29
	%	8.1	10.9	4.5	1.6	1.2	7.3	0.4	2.8	7.3	11.7
Almost never	*n*	59	59	31	13	26	68	24	56	54	76
	%	23.9	23.9	12.6	5.3	10.5	27.5	9.7	22.7	21.9	30.8
Sometimes	*n*	102	90	101	82	99	99	105	90	105	81
	%	41.3	36.4	40.9	33.2	40.1	40.1	42.5	36.4	42.5	32.8
Fairly often	*n*	52	53	65	101	102	45	96	76	51	41
	%	21.1	21.5		26.3	41.3	18.2	38.9	30.8	20.6	16.6
Always	*n*	14	18	39	47	17	17	21	18	19	20
	%	5.7	7.3	15.8	19.0	6.9	6.9	8.5	7.3	7.7	8.1

**FIGURE 1:**
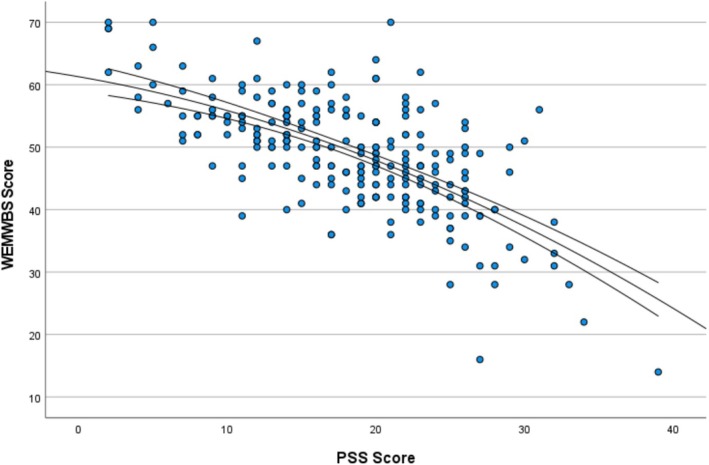
WEMWBS scores plotted against PSS scores. A quadratic line fit and 95% confidence interval are shown. A strong negative trend is seen (Spearman's correlation coefficient = –0.620; *p* < 0.001).

As shown in Figure 1, WEMWDS scores and PSS scores were found to be (fairly) moderate to strongly negatively correlated (Spearman's correlation coefficient = −0.620; *p* < 0.001, 95% confidence interval: −0.693 to −0.534). This means that on average, as perceived stress increased, wellbeing was found to decrease.

**FIGURE 2:**
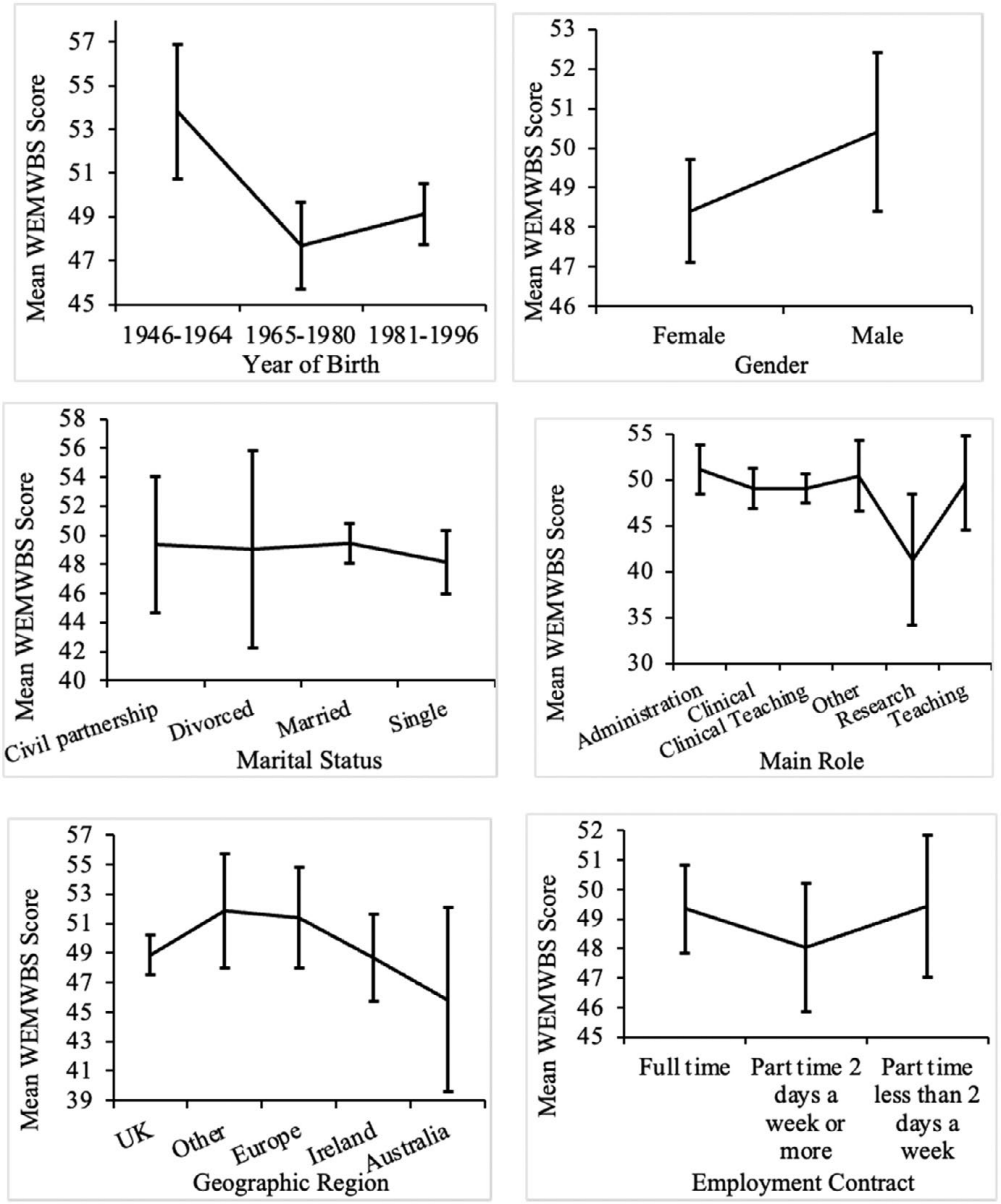
Results for the mean (WEMWBS) wellbeing score presented for: (top left) year of birth; (top right) gender; (middle lead) marital status; (middle right) main role; (bottom left) geographic region; (bottom right) employment contract (Error bars indicate 95% confidence intervals of the mean).

**FIGURE 3:**
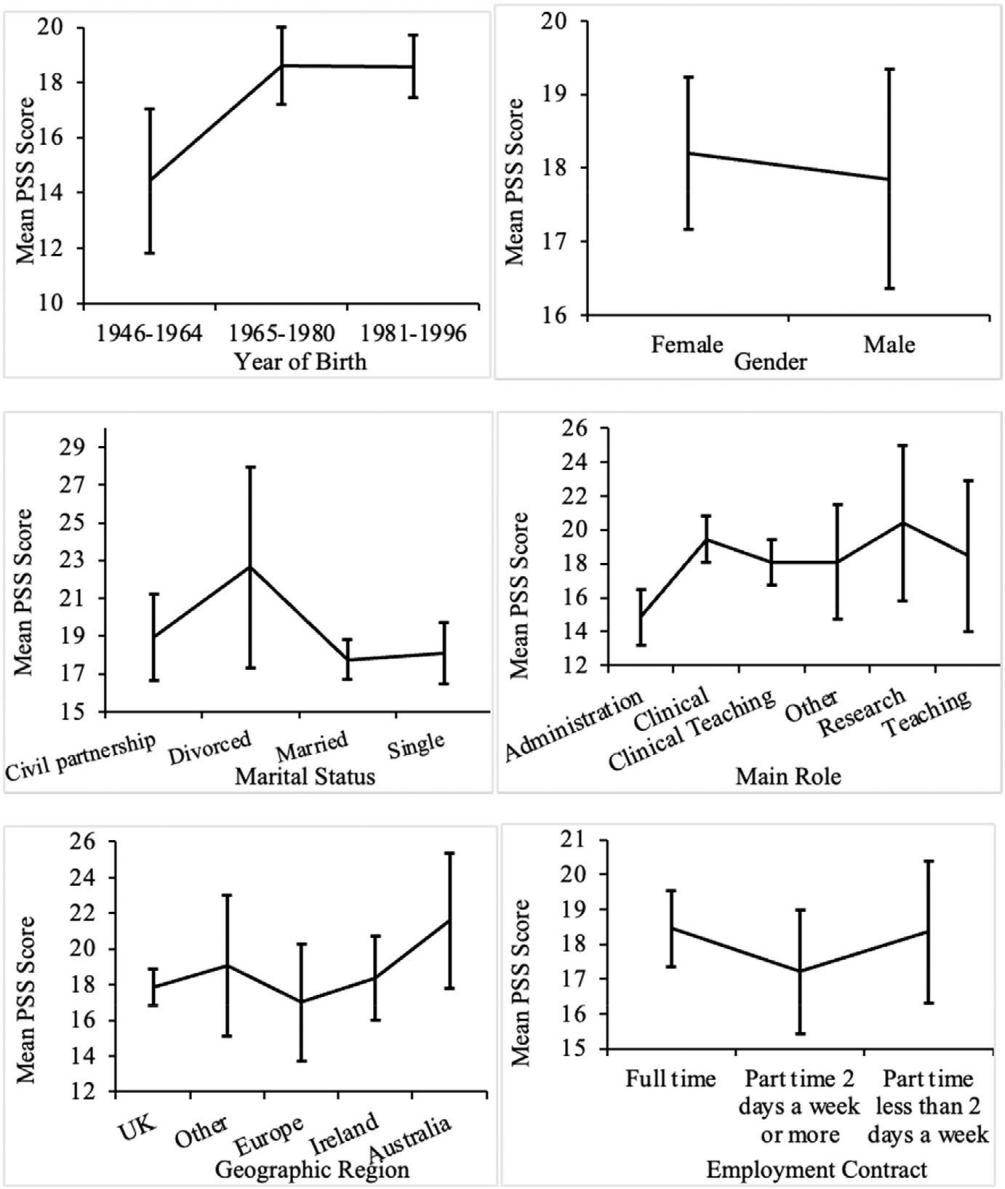
Results for the mean (PSS) stress score presented for: (top left) year of birth; (top right) gender; (middle lead) marital status; (middle right) main role; (bottom left) geographic region; (bottom right) employment contract (Error bars indicate 95% confidence intervals of the mean).

WEMWBS scores with 95% confidence intervals are shown as a function of the main factors in Figure 2. Strong effects were observed only on year of birth (mixed model: *p* = 0.013) and work role (mixed model: *p* = 0.013). Year of birth for 1997–2012 and gender identities other than male and female were excluded from analysis due to very small sample sizes (Table 4). The mean PSS scores with 95% confidence intervals are shown as a function of the main factors in Figure 3. Strong effects were observed only on year of birth (mixed model: *p* = 0.019) and work role (mixed model: *p* = 0.029). Year of birth for 1997–2012 and gender identities other than male and female were excluded from analysis due to very small sample sizes (Table 4). Results of the mixed‐effects models, including regression coefficients and associated confidence intervals, are presented in more detail in Tables A1 and A2.

**TABLE 4:** Results for WEMWBS scores and PSS scores as a function of year of birth, gender, geographic region, marital status, employment and role.

**Year of birth**	1946–1964	1965–1980	1981–1996				Univariate statistical test, *p*	Mixed Model, *p*	Effect size
WEMWBS score	53.8 (7.85) 53.0	47.8 (9.75) 50.0	49.1 (7.69) 50.0				0.005	0.013	Moderate
PSS score	14.4 (6.62) 14.0	18.53 (6.99) 19.0	18.6 (6.23) 19.5				0.009	0.019	Moderate
**Gender**	Female	Male							
WEMWBS score	48.4 (8.60) 49.0	50.4 (8.88) 50.0					0.097	0.133	Weak
PSS score	18.2 (6.75) 19.0	17.9 (6.57) 19.0					0.697	0.774	None
**Geographic region**	UK	Other	Europe	Ireland	Australia				
WEMWBS score	48.9 (9.00) 50.0	51.9 (6.72) 53.5	51.4 (6.17) 51.0	48.7 (8.62) 48.0	46.9 (9.60) 48.0		0.37	0.691	Weak/Moderate
PSS score	17.9 (6.72) 19.0	19.1 (9.87) 19.0	17.0 (5.94) 16.0	18.3 (6.86) 17.0	21.2 (6.13) 20.0		0.38	0.512	Weak
**Marital status**	Civil Partnership	Divorced	Married	Single					
WEMWBS score	49.4 (6.96) 48.0	49.0 (10.1) 51.0	49.5 (8.47) 50.0	48.2 (9.30)			0.793	0.839	Weak
PSS score	18.9 (3.39) 19.0	22.6 (7.90) 22.0	17.7 (6.54) 18.0	18.1 (6.96) 19.0			0.129	0.065	Moderate
**Employment**	Full time	Part time 2 days a week or more	Part time < 2 days a week						
WEMWBS score	49.4 (9.08) 50.0	48.0 (8.66) 47.5	49.4 (7.25) 50.0				0.574	0.537	Weak
PSS score	18.4 (6.63) 19.0	17.2 (7.10) 17.5	18.4 (6.09) 20.0				0.454	0.438	Weak
**Role**	Administration	Clinical	Teaching (Clinical)	Other	Research	Teaching (Non‐Clinical)			
WEMWBS score	51.1 (7.53) 52.0	49.0 (8.18) 50.0	49.1 (8.28) 50.0	50.4 (8.37) 51.0	41.8 (13.2) 46.0	49.7 (9.27) 47.0	0.013	0.02	Moderate/Strong
PSS score	14.8 (4.59) 14.5	19.5 (5.19) 20.0	18.1 (7.05) 19.0	18.1 (7.40) 19.0	20.0 (8.49) 18.0	18.5 (8.05) 20.0	0.037	0.029	Moderate

*Note:* Results shown in each element of the table are: Mean, (standard deviation) and median. (Europe: Excluding the UK and Ireland. Year of birth for 1997–2012 and gender identities other than male and female were excluded from analysis due to very small sample sizes). Results are shown also for each factor for *p*‐values from a univariate statistical test (one‐way ANOVA), a mixed model (adjusting for confounding), as well as a description of the effect size of each factor established by finding eta^2^ (an effect size measure available in SPSS V29 for parametric ANOVA) and independently also via Cohen's *d*.

The main research questions of this study related to how wellbeing measured via the WEMWBS scores and perceived stress measured via the PSS scores varied with respect to the levels of the main factors, namely year of birth, gender, marital status, work role, geographic region and employment (full time versus part time) (Figures 2 and 3). Table 4 also summarises an analysis of absolute effect sizes (established via eta squared values for ANOVA and also an estimate of Cohen's *d*), as well as presenting results of univariate statistical tests and also a ‘mixed’ model that adjusts for potentially confounding influences.

Two factors, in particular, showed moderate to strong effect sizes that were statistically significant for the primary outcomes, namely, year of birth and work role. Figure 2 shows that those subjects who were born in years 1946 to 1964 demonstrated significantly higher levels of wellbeing established via the WEMWBS scores compared to 1946–1964 (Tukey's post hoc test, *p* = 0.003) and 1981–1996 (Tukey's post hoc test, *p* = 0.031). Similarly, Figure 3 shows that perceived stress measured via PSS scores is much lower for those subjects who were born in years 1946–1964 compared to 195 to 1980 (Tukey's post hoc test, *p* = 0.011) and 1981 to 1996 (Tukey's post hoc test, *p* = 0.010). Figure 2 indicates also that researchers had lower wellbeing compared to other roles, in particular: administrative, clinical, clinical teaching and other roles (Tukey's post hoc test for WEMWBS scores, *p* < 0.05). By contrast, perceived stress from PSS scores shown in Figure 3 seemed lower for administrative roles compared to other roles, in particular: clinical and research roles (Tukey's post hoc test for PSS scores, *p* < 0.05). There was a weak effect with respect to gender, with males having slightly higher levels of wellbeing via WEMWBS scores and lower levels of perceived of stress measured via PSS scores compared to females (mixed model: *p* > 0.05). (Gender identities other than male and female were excluded from analysis due to very small sample sizes.) Effects of marital status were weak and not statistically significant (mixed model: *p* > 0.05) for wellbeing measured via WEMWBS scores. However, there was some evidence that divorced subjects had higher levels of stress via PSS scores (mean = 22.6, median = 22.0, SD = 7.90), although again this was not statistically significant (mixed model: *p* > 0.05). Working status (i.e., part time versus full time) did not seem to affect either wellbeing measured via WEMWBS scores in Figure 2 or perceived levels of stress measured via PSS scores in Figure 3 (for both WEMWBS and PSS scores, mixed model: *p* > 0.05). Finally, there was some evidence that wellbeing measured was lower and perceived levels of stress higher for Australia versus other geographic regions. However, this was a weak effect (from effects sizes) for both variables and was not statistically significant in either case (mixed model: *p* > 0.05; note again that region was modelled as a random effect in our ‘mixed model’).

Wellbeing support needs were explored in this survey, where 132 subjects out of 247 said that they felt that they had needed some form of support for their wellbeing in the past. Table 5 shows the types of wellbeing accessed by respondents previously. We see that meetings with line manager or a senior colleague were the most common forms of support accessed. For example, 36 out of 247 subjects (14.6%) responded ‘Often’ or ‘Very Often’ to accessing informal meetings. As shown in Table 2, respondents accessed all other types of support only occasionally at best. The number of people who responded ‘Sometimes’, ‘Often’ or ‘Very Often’ for other types of support was: formal meetings (*n* = 57; 23.1%), self‐help resources (*n* = 47; 19.0%) and wellbeing workshops or seminars (*n* = 44; 17.8%). Finally, access to wellbeing services was examined in the survey also. Thirty‐seven respondents out of 247 (15.0%) said that they were unaware of any wellbeing services or facilities, whereas 35 (14.2%) that they were unsure how to seek help. Eighteen subjects (7.3%) said access or location was inconvenient, 9 (3.6%) said that long waiting lists were an issue, and 51 (20.6%) said that they were unsure if the services or facilities that are available can be of help to them. Forty‐eight subjects (19.4%) responded that they had no need for the types of services/facilities that are available, whereas 55 (22.3%) said that they had no time to use the services/facilities.

**TABLE 5:** Types of wellbeing support accessed by respondents.

		Staff helpline	Contacting key wellbeing contacts	Self‐help resources	Referral for wellbeing counselling	Wellbeing workshops/seminars	Signposting to external support services	Formal meetings with line manager/senior colleague	Informal meeting with line manager/senior colleague
Never	*n*	225	227	186	216	185	213	170	110
	%	91.1	91.9	75.3	87.4	74.9	86.2	68.8	44.5
Almost never	*n*	13	12	14	15	18	10	20	38
	%	5.3	4.9	5.7	6.1	7.3	4.0	8.1	15.4
Sometimes	*n*	7	5	38	11	34	16	46	63
	%	2.8	2.0	15.4	4.5	13.8	6.5	18.6	25.5
Often	*n*	2	2	6	3	9	3	7	28
	%	0.8	0.8	2.4	1.2	3.6	1.2	2.8	11.3
Very often	*n*	0	1	3	2	1	5	4	8
	%	0.0	0.4	1.2	0.8	0.4	2.0	1.6	3.2

## 4 Discussion

The aim of this study was to determine the stress and wellbeing of the staff involved in dental education and identify any barriers they face in accessing wellbeing services. In addition, it has highlighted how wellbeing and perceived stress varied with respect to year of birth, gender, marital status, work role, geographic region and employment status (full‐time vs. part‐time).

Studies have shown that mental wellbeing and stress are reported by dental professionals worldwide, including in countries such as Australia [19], USA [20] and Canada [21]. The mean WEMWDS score of participants in this study was 49.0, which is slightly lower than that reported in the general population in the UK (51.0), Spain (58.1) and Denmark 52.2 [22, 23], but similar to Scotland [24]. The mean PSS score in this study was 18.1, indicating moderate levels of stress; this is consistent with other studies measuring stress in dental professionals who reported moderate levels of stress in a sample of dentists [25], but it was more than that of the general population in the UK [26]. Unsurprisingly, PSS and WEMWDS were negatively correlated, indicating that increased levels of perceived stress led to lower levels of wellbeing. Similar results have been seen in university students, with perceived stress reported to have a major impact on students' mental wellbeing [27]. The results of this study showed that gender, marital status, geographical location and employment type had little to no effect on the perceived stress or levels of wellbeing of those working within dental education.

### 4.1 Age

Results of this study show that levels of wellbeing are significantly higher and reported stress scores significantly lower for participants born in 1946–1964 when compared to the younger age categories. These findings are similar to the results of other studies that have measured perceived stress across different age groups and show that adults of different ages perceive varying amounts of stress [28, 29]. There is limited literature on studies reporting WEMWBS scores for different age groups. However, a study of higher education staff in Portuguese Institutions found that university teachers over 60 years old and those with more than 30 years of teaching experience exhibited lower levels of perceived stress [30]. Another study revealed that younger dental faculty members reported statistically significantly lower levels of wellness [16].

The elevated levels of perceived stress reported in younger participants may be related to stress perception. Younger participants may perceive work stressors as more stressful due to a lack of ‘coping strategies’ or resilience. Resilience describes a better‐than‐expected outcome despite adversity or setbacks. Studies on reporting higher levels of resilience to have beneficial effects on such stress perceptions and their responses [31].

Older participants in this study may have more experience in their job roles, meaning that they are better able to manage and control their daily workload or were more strategic and thus less susceptible to stress and poor wellbeing. Their past experiences may have resulted in participants developing strategies to overcome stress. Hertel et al. have described how older workers report more active problem‐focused coping whereas younger workers report more avoidance [32]. Problem‐focused coping strategies involve confrontation with a problem to manage it. An example of this is writing a list of specific tasks to complete, allowing prioritisation of tasks to minimise stress. The level of stress a person experiences is directly linked to how confident they feel about dealing with the threat [4]. Staff working in dental education must balance numerous job roles and responsibilities which may lead to elevated levels of stress. However, if they are equipped with strategies to manage their stress, they are more likely to have higher levels of wellbeing.

All staff, but particularly those with less experience, would benefit from training on the development of coping strategies to decrease levels and perceived stress and improve overall wellbeing.

### 4.2 Work Role

Staff working as researchers reported the lowest wellbeing when compared to other roles within dental education. This may be a result of the stresses of keeping and maintaining funding for research projects and no guarantee for long‐term, stable employment [33]. In this study, the perceived stress score was the lowest, and WEMWBS was highest for those working in non‐teaching administrative roles. Other studies report that academics have higher levels of stress and poorer work‐life balance than their non‐academic colleagues [34, 35]. Staff working in academia may report higher stress levels and a poorer quality of life due to the need to work extra hours and poorer work‐life balance [36].

### 4.3 Barriers to Accessing Wellbeing Support

Despite the availability of wellbeing services in many institutions, the uptake among dental educators remains low. Over 50% of respondents who needed support did not access the available services, citing several barriers. This mainly included lack of awareness (15%), uncertainty about the effectiveness of services (20.6%) and time constraints (22.3%). This is consistent with anecdotal reports and previous research suggesting that, while wellbeing services are often available, their utility is hindered by poor engagement and limited awareness among staff [13].

Interestingly, many respondents preferred informal meetings with line managers or senior colleagues as a form of support, rather than formal wellbeing services or self‐help resources. This preference for peer support may indicate that dental educators value personal interaction and guidance in managing stress over institutional or formal interventions. However, it also highlights a gap in the perceived accessibility and effectiveness of existing services. Institutions may need to rethink how they promote and structure wellbeing initiatives, making them more approachable, flexible and tailored to the specific needs of their staff. In addition, it would be valuable to train line managers in identifying staff with poor wellbeing; supporting them or signposting to get prompt and appropriate help would be beneficial to both the individual staff member and the organisation.

### 4.4 Implications for Educational Institutions

Educational institutions have a duty of care and a moral responsibility to create supportive environments that promote the mental health and wellbeing of their staff. The findings from this study suggest that existing support mechanisms may not be fully meeting the needs of dental educators, particularly those in research, clinical roles, or younger faculty members. Institutions should consider adopting appropriate wellbeing services to enhance staff wellbeing, enhancing the visibility of their wellbeing services, ensuring that staff are both aware of and comfortable with accessing these resources. Addressing barriers such as time constraints and perceived ineffectiveness may also help to improve engagement.

Furthermore, targeted interventions should be developed for younger educators and those in more stressful roles, such as clinical teaching positions. These might include mentorship programmes, workload management strategies and resilience training. Given the critical link between educator wellbeing and professional performance, improving mental health support can have far‐reaching benefits, including better patient care and reduced absenteeism [15].

Dental educators suffering from stress and low wellbeing will only have a detrimental impact on themselves, but they may not be able to effectively deliver the curriculum and nurture their students to develop into safe and competent dental professionals.

## 5 Limitations

A convenience sample was used for this study; however, that means that it may not be representative of the population at large. Therefore, there may be limited external validity, as the findings cannot easily be generalised to populations with characteristics that differ from the population that was conveniently accessible and from which the sample was drawn [37].

The survey was accessible to all staff who are involved in dental education and was advertised via a newsletter so the response rate of the survey could not be calculated. The survey was written in English, which may not have been the first language of a few participants. However, English is the agreed language of communication at the ADEE.

## 6 Conclusion

Staff involved in dental education report higher stress and lower wellbeing levels than the general population. They face barriers to accessing wellbeing services their educational organisations may provide them and are more likely to approach their line manager or senior colleague to seek help. Those in the younger age group and in job roles such as research and clinical teaching are more affected. It is vital that educational establishments establish strategies to promote the wellbeing of their staff members and improve access to services to ensure continued high‐quality training of our future dental workforce. Further exploration of the factors influencing staff wellbeing and suggestions for improvement are needed to help educational providers develop effective strategies.

## Conflicts of Interest

The authors declare no conflicts of interest.

## Data Availability

The data that support the findings of this study are available from the corresponding author upon reasonable request.

## Appendix A The Warwick‐Edinburgh Mental Wellbeing Scale (WEMWBS)

**Table:**

Part 1 is based on Warwick‐Edinburgh Mental Wellbeing Scale (WEMWBS)	Scoring (1–5)				
Please tick the box that best describes your experience of each over the last 2 weeks	None of the time	Rarely	Some of the time	Often	All of the time
I've been feeling optimistic about the future	1	2	3	4	5
I've been feeling useful	1	2	3	4	5
I've been feeling relaxed	1	2	3	4	5
I've been feeling interested in other people	1	2	3	4	5
I've had energy to spare	1	2	3	4	5
I've been dealing with problems well	1	2	3	4	5
I've been thinking clearly	1	2	3	4	5
I've been feeling good about myself	1	2	3	4	5
I've been feeling close to other people	1	2	3	4	5
I've been feeling confident	1	2	3	4	5
I've been able to make up my own mind about things	1	2	3	4	5
I've been feeling loved	1	2	3	4	5
I've been interested in new things	1	2	3	4	5
I've been feeling cheerful	1	2	3	4	5

## Appendix B Perceived Stress Scale (PSS‐10)

**Table:**

Part 2 b** In context of your workplace, please tick the box that best describes your experience about your feelings and thoughts during the last month	Scoring (0–4)				
	Never	Almost never	Sometimes	Fairly often	Very often
In the last month, how often have you been upset because of something that happened unexpectedly?	0	1	2	3	4
In the last month, how often have you felt that you were unable to control the important things in your life?	0	1	2	3	4
In the last month, how often have you felt nervous and ‘stressed’?	0	1	2	3	4
In the last month, how often have you felt confident about your ability to handle your personal problems?	0	1	2	3	4
In the last month, how often have you felt that things were going your way?	0	1	2	3	4
In the last month, how often have you found that you could not cope with all the things that you had to do?	0	1	2	3	4
In the last month, how often have you been able to control irritations in your life?	0	1	2	3	4
In the last month, how often have you felt that you were on top of things?	0	1	2	3	4
In the last month, how often have you been angered because of things that were outside of your control?	0	1	2	3	4
In the last month, how often have you felt difficulties were piling up so high that you could not overcome them?	0	1	2	3	4

## Appendix C

**TABLE A1:** Parameter estimates from the mixed‐effects model using WEMWDS score as the dependent variable.

	Class	Parameter	SE	*t*	*p*	95% confidence interval	
						Lower bound	Upper bound
Intercept		49.403	3.694	13.376	< 0.001	42.124	56.682
Year of study	1946–1964	4.315	1.917	2.251	0.025	0.538	8.092
	1965–1980	−1.389	1.254	−1.108	0.269	−3.861	1.083
	1981–1996	Reference class					
Gender	Female	−1.892	1.253	−1.509	0.133	−4.362	0.578
	Male	Reference class					
Marital status	Civil Partnership	0.094	2.915	0.032	0.974	−5.652	5.839
	Divorced	0.028	2.860	0.010	0.992	−5.607	5.664
	Married	1.128	1.332	0.847	0.398	−1.496	3.753
	Single	Reference class					
Employment	Full time	−0.500	1.659	−0.302	0.763	−3.770	2.769
	Part time 2 days a week or more	−1.810	1.826	−0.991	0.323	−5.409	1.789
	Part time < 2 days a week	Reference class					
Role	Administration	0.917	2.802	0.327	0.744	−4.604	6.439
	Clinical	−0.615	2.617	−0.235	0.814	−5.772	4.542
	Clinical Teaching	−0.887	2.447	−0.363	0.717	−5.708	3.934
	Other	0.360	2.980	0.121	0.904	−5.513	6.232
	Research	−8.810	3.254	−2.707	0.007	−15.223	−2.396
	Teaching (non‐clinical)	Reference class					
Geographical region	UK	1.891	2.834	0.667	0.505	−3.693	7.475
	Other	4.879	3.650	1.337	0.183	−2.314	12.073
	Europe	2.878	3.495	0.823	0.411	−4.010	9.766
	Ireland	1.736	3.231	0.537	0.592	−4.630	8.102
	Australia	Reference class					

*Note:* The general pattern of results for the parameter estimates with respect to the categories for each of the variables agrees with those results for means presented in Table 4.

**TABLE A2:** Parameter estimates from the mixed–effects model using PSS score as the dependent variable.

	Class	Parameter	SE	*t*	*p*	95% confidence interval	
						Lower bound	Upper bound
Intercept		20.512	2.816	7.284	< 0.001	14.962	26.061
Year of study	1946–1964	−3.963	1.461	−2.712	0.007	−6.843	−1.083
	1965–1980	−0.052	0.956	−0.054	0.957	−1.936	1.833
	1981–1996	Reference class					
Gender	Female	0.275	0.956	0.288	0.774	−1.608	2.158
	Male	Reference class					
Marital status	Civil Partnership	0.901	2.223	0.405	0.686	−3.480	5.282
	Divorced	5.720	2.181	2.623	0.009	1.423	10.017
	Married	0.293	1.016	0.288	0.773	−1.708	2.294
	Single	Reference class					
Employment	Full time	1.204	1.265	0.952	0.342	−1.289	3.697
	Part time 2 days a week or more	0.026	1.393	0.019	0.985	−2.718	2.770
	Part time < 2 days a week	Reference class					
Role	Administration	−3.463	2.136	−1.621	0.106	−7.672	0.747
	Clinical	1.617	1.995	0.810	0.419	−2.315	5.549
	Clinical Teaching	−0.007	1.865	−0.004	0.997	−3.683	3.669
	Other	0.027	2.272	0.012	0.990	−4.451	4.505
	Research	2.126	2.481	0.857	0.393	−2.764	7.016
	Teaching (non‐clinical)	Reference class					
Geographical region	UK	−3.650	2.160	−1.690	0.093	−7.908	0.607
	Other	−2.384	2.783	−0.857	0.393	−7.869	3.100
	Europe	−3.627	2.665	−1.361	0.175	−8.879	1.625
	Ireland	−3.193	2.463	−1.296	0.196	−8.047	1.662
	Australia	Reference class					

*Note:* The general pattern of results for the parameter estimates with respect to the categories for each of the variables agrees with those results for means presented in Table 4.
